# Novel Anti-Nicotine Vaccine Using a Trimeric Coiled-Coil Hapten Carrier

**DOI:** 10.1371/journal.pone.0114366

**Published:** 2014-12-10

**Authors:** Keith D. Miller, Richard Roque, Christopher H. Clegg

**Affiliations:** TRIA Bioscience Corp, Seattle, Washington, United States of America; Tulane University, United States of America

## Abstract

Tobacco addiction represents one of the largest public health problems in the world and is the leading cause of cancer and heart disease, resulting in millions of deaths a year. Vaccines for smoking cessation have shown considerable promise in preclinical models, although functional antibody responses induced in humans are only modestly effective in preventing nicotine entry into the brain. The challenge in generating serum antibodies with a large nicotine binding capacity is made difficult by the fact that this drug is non-immunogenic and must be conjugated as a hapten to a protein carrier. To circumvent the limitations of traditional carriers like keyhole limpet hemocyanin (KLH), we have synthesized a short trimeric coiled-coil peptide (TCC) that creates a series of B and T cell epitopes with uniform stoichiometry and high density. Here we compared the relative activities of a TCC-nic vaccine and two control KLH-nic vaccines using Alum as an adjuvant or GLA-SE, which contains a synthetic TLR4 agonist formulated in a stable oil-in-water emulsion. The results showed that the TCC's high hapten density correlated with a better immune response in mice as measured by anti-nicotine Ab titer, affinity, and specificity, and was responsible for a reduction in anti-carrier immunogenicity. The Ab responses achieved with this synthetic vaccine resulted in a nicotine binding capacity in serum that could prevent >90% of a nicotine dose equivalent to three smoked cigarettes (0.05 mg/kg) from reaching the brain.

## Introduction

Tobacco addiction is the second leading cause of death in the world and the single largest cause of cancer and heart disease. Currently there are 1.3 billion tobacco smokers worldwide resulting in an estimated 6 million deaths a year, and given present smoking trends, tobacco will kill 10 million people each year by 2020 [1]. Tobacco use is the leading preventable cause of death in the United States. Each year nearly half of the 42 million adult smokers attempt to quit, yet due to the highly addictive nature of nicotine, less than 5% succeed [2]. Aids to smoking cessation include supportive counseling, nicotine replacement (gums, patches, etc.), and receptor antagonists that reduce nicotine reward and withdrawal symptoms. Unfortunately, long-term outcomes for nicotine replacement therapies remain poor and achieve an abstinence rate of only 10–20% after the first year [3]–[5].

Anti-addiction vaccines induce antibodies that block the pharmacological effects of drugs like nicotine [6]. To date, vaccines for smoking cessation have shown considerable promise in preclinical animal models for their ability to diminish nicotine-mediated physiological and behavioral responses including nicotine craving [7]–[9]. However, clinical studies in humans failed to measure significant differences in smoking abstinence between the intervention and placebo groups [10], [11]. Importantly, subgroup analyses from two Phase II studies indicated that subjects with the highest antibody (Ab) titers showed increased abstinence at 12 months, and the non-abstaining subjects within the high Ab group reduced daily cigarette consumption by 50%. Encouraged by these findings, two subsequent Phase 3 studies attempted to increase Ab titers even further by modifying vaccine dose and immunization schedule. However, clinical responses were not improved and both studies failed to achieve the established efficacy endpoints. To investigate this result further, a follow-on study measured the nicotine binding capacity induced in vaccinated subjects and determined that the Abs could only prevent ∼12% of an infused dose of nicotine from reaching the brain [12]. Thus, the resulting Ab responses induced by the vaccine were insufficient for providing clinical benefit. This has raised questions about the requirements for improved vaccine efficacy.

Nicotine and other drugs of abuse are non-immunogenic and must be conjugated to a protein carrier to facilitate antigen presentation and the induction of T cell help, which regulates durable Ab responses and memory generation. To date, most hapten carriers are derived from microbial sources like keyhole limpet hemocyanin (KLH), tetanus toxoid, diphtheria toxoid, and pseudomonas exotoxin A [8], [9], [11], [13], although they may be limited in important ways. For instance, epitope density is a critical factor influencing the magnitude and quality of the immune response [14]–[20]. However, the maximum number of haptens that can be loaded on the clinical candidate vaccines, which is typically dictated by the number of lysines used for chemical conjugation, is less than 50. Also, hapten stoichiometry and spacing likely varies within each carrier and uncertainty remains about which linkages within the protein present the best epitope for stimulating high affinity Ab titers. Another potential problem is that these proteins are highly immunogenic and might induce anti-carrier antibodies that could limit booster immunizations over time. “Epitopic suppression” is a widely recognized phenomenon first observed with licensed polysaccharide conjugate vaccines, and experiments have shown that this effect is suppressed by increasing hapten density [21]–[23].

To circumvent the limitations of current anti-nicotine vaccines, we have produced a synthetic hapten carrier using a short trimeric coiled-coil peptide (TCC) comprising three amphipathic α-helices rich in lysines for increasing epitope density. The C-terminal portion of each monomer peptide also contains CD4 T cell epitopes required for the production of long-lived plasma cells and high affinity antibodies [24], [25]. Here we describe the activity of a TCC-based nicotine vaccine that was formulated with two different adjuvants; Alum and GLA-SE a two part adjuvant formulation containing the synthetic TLR4 agonist Glucopyranosyl Lipid Adjuvant (GLA) and a stable oil-in-water emulsion (SE) [26], [27]. The results demonstrate that the adjuvanted TCC hapten carrier induces a very effective immune response in mice as measured by anti-nicotine Ab titer, specificity, affinity, and function.

## Materials and Methods

### Ethics Statement

This study was carried out in strict accordance with the recommendations in the Guide for the Care and Use of Laboratory Animals of the National Institutes of Health, the US Public Health Service Policy on Humane Care and Use of Laboratory Animals, and the Association for Assessment and Accreditation of Laboratory Animal Care International (AAALAC). Protocol #2012-8 was approved by the Institutional Animal Care and Use Committees of the Infectious Disease Research Institute which operates under a currently approved Assurance #A4337-01 and USDA certificate #91-R-0061. Animal welfare and health was monitored daily and in instances where medical intervention was not effective, animals were humanely euthanized and every effort was made to minimize suffering.

### Molecular Modelling

**Figure 1:**
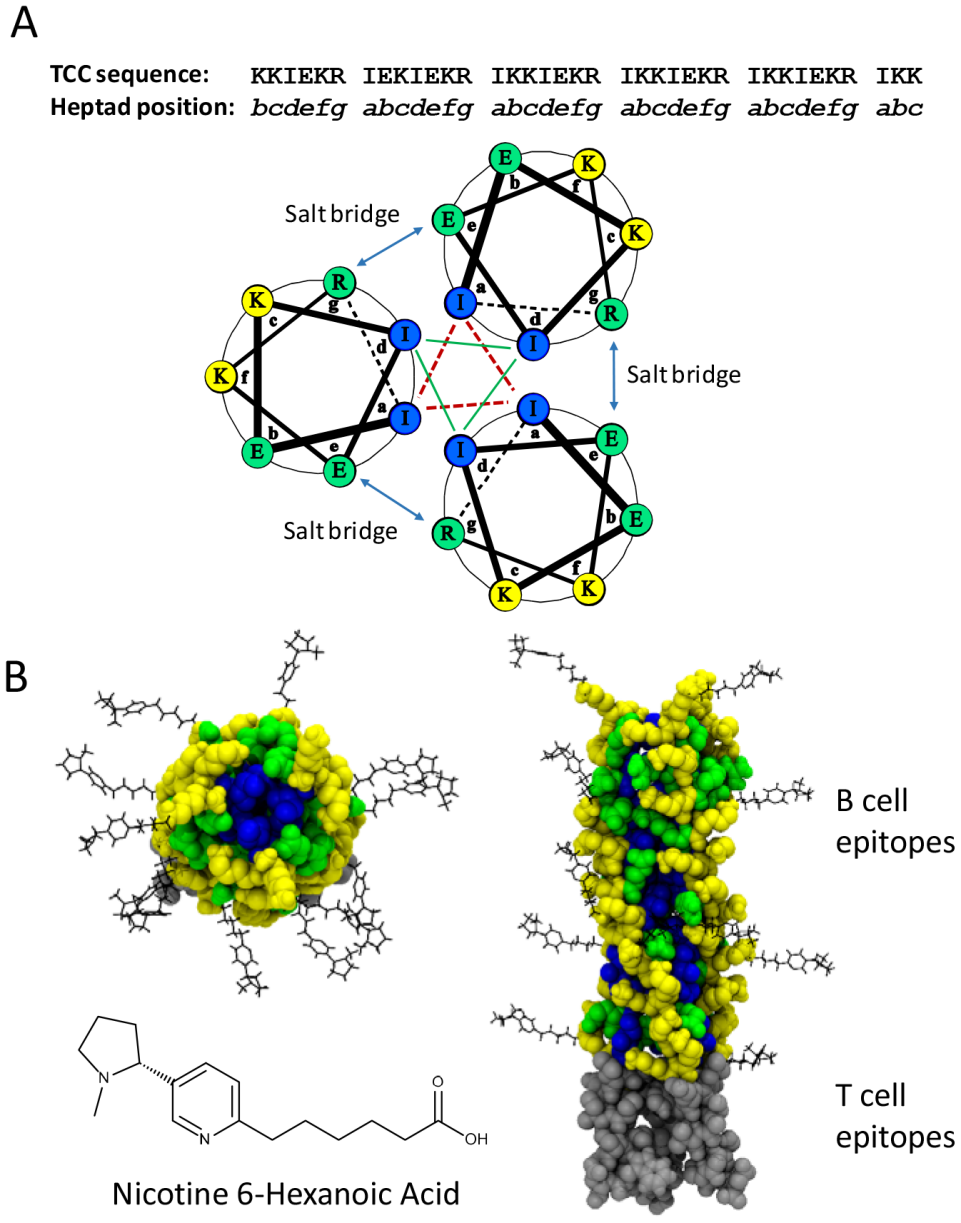
Structure of the Trimeric Coiled Coil protein. (A) The amino acid sequence of each amphipathic peptide is comprised of 5 heptad repeats containing isoleucine at the “a” and “d” positions to form the hydrophobic interior of the trimer and lysine, arginine, and glutamic acid at the remaining positions. The carboxyl sequences of the CD4 T cell epitope are not represented. The position of each residue within the heptad repeat following self-assembly of the trimeric protein is represented by the three helical wheel projections. Forty eight lysine residues (yellow) are solvent exposed and available for hapten conjugation. Isoleucines (blue) comprise the hydrophobic core and arginine and glutamic acid residues (green) form salt bridges that stabilize the trimeric structure. (B) Homology model of the TCCnic peptide with rendered atoms represented by space filling Van der Waals radii. The model on the left is a view down the symmetrical axis of the trimer, whereas the right-hand model is a longitudinal view. The C-terminal portion contains CD4 T-cell epitopes (grey spheres) required for B-cell help. Nicotine-6-hexanoic acid haptens (black bonds) were manually added to the ε-amino group of 12 randomly chosen lysine residues using the Molefacture plug-in from the Visual Molecular Dynamics software package. Further details of model construction are provided in the “Materials and Methods” section.

The structure of the TCC peptide was obtained by homology modelling using the SWISS-MODEL software [28] and a trimeric autotransporter adhesion fragment coiled-coil motif [29] as the template. The resulting structure had a GMQE score of 0.75 and QMEAN4 score of 1.34 and was minimized after manually adding the T-cell epitope residues using the SwissPDBViewer 4.1.0 software package [30]. Nicotine-6-hexanoic acid was assembled in MarvinSketch 6.1.3 (ChemAxon LLC, Cambridge, MA) and the lowest energy conformer saved as a PDB file. The nicotine-6-hexanoate conformer was added to the ε-amino group of 12 randomly chosen lysine residues of the TCC peptide using the Molefacture plug-in (version 1.3) of the Visual Molecular Dynamics software package [31] and rendered using the embedded Tachyon ray tracing software (Fig. 1).

### Antigens

The TCC was synthesized by standard solid-phase coupling methodologies and dissolved in PBS for conjugation and immunizations (Biosynthesis Inc., Lewiston, TX). To increase serum half-life, the alpha N-terminal lysine was acetylated and used without de-protection. Keyhole limpet hemocyanin (KLH) was purchased from Sigma-Aldrich (St. Louis, MO) and resuspended according to the manufacturer's protocol. The Nic-6-HA hapten, which contains hexanoic acid at the 6-position of the nicotine pyridine ring, was synthesized using nicotine modified by bromination (Medchem Source, Federal Way WA), followed by coupling to the hexanoic acid w-alkynyl ester, reduction to the alkane, and deprotection to the free acid. The N-hydroxysuccinimide (NHS) ester of Nic-6-HA was synthesized in anhydrous methanol containing 1.3 equivalents each of NHS (dissolved in anhydrous DMF) and N-(3-dimethylaminopropyl)-N′-ethylcarbodiimide hydrochloride (EDC; dissolved in anhydrous ethanol). After 45 min at room temperature, the solution was adjusted to 10 mM DTT (dissolved in anhydrous ethanol) and incubated for 5 min at room temperature to inactivate excess EDC. Increasing molar equivalents of (Nic-6-HA)-NHS ester were added to TCC or KLH and incubated for 30 min at room temperature. TCC-nic was desalted into PBS using Bio-Gel P2 polyacrylamide resin (800–1,800 MW fractionation range; Bio-Rad Laboratories, Inc. Hercules, CA), and KLH-Nic was desalted into PBS +0.75 M NaCl using 7,000 MWCO Zebra spin columns (Thermo Fisher Scientific, Rockford, IL). Previous studies have shown an excellent correlation between UV spectroscopy and mass spectrometry based hapten quantification methods [32], [33], so after desalting, the UV absorbance of the TCC-Nic peptides were measured from 220–320 nm. Nicotine conjugation to each carrier was quantified as previously described [34], using extinction coefficients at A280 nm calculated by the ProtParam software program [35] for the hapten carriers (e.g. 25,440 M^−1^ cm^−1^ at 280 nm and 20,274 M^−1^ cm^−1^ at 264 nm for the TCC peptide), and a molar extinction coefficient of 2,959 M^−1^ cm^−1^ for nicotine-6-HA at 264 nm and 270 M^−1^ cm^−1^ at 280 nm. Thus, to calculate the average nicotine:TCC peptide molar ratios, the absorbance of TCCnic was measured at 264 and 280 nm and the values inserted into the equation described by Hamblett *et al.* [34]. For example, the TCC peptide had an A264:A280 ratio of 3.94, so the calculated nicotine peptide ratio would be: 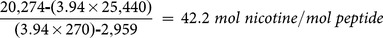



Prior to immunizations, all antigens were processed with the ToxinEraser Endotoxin Removal Kit (GenScript, Piscataway, NJ) according to the manufacturer's protocol.

### Immunizations

Female C57BL/6 mice (The Jackson Laboratory, Bar Harbor ME) were maintained under pathogen-free conditions and all experimental protocols were conducted in accordance with the guidelines of the Infectious Disease Research Institute (IDRI, Seattle WA). The hapten-conjugated TCC and KLH carriers were combined on the day of immunization with either a 1∶1 dilution of 40 mg/ml Imject Alum Adjuvant (Thermo Scientific, Rockford, IL) or with GLA-SE containing 5 ug of the synthetic TLR4 agonist, GLA, which is formulated in a final 2% oil-in-water stable emulsion [26]. Mice were injected with 50 ul in each hind quadriceps muscle (100 ul final volume) on days 0, 14, 131 and serum was collected on days 14, 28, 82, 138, and 160 for measuring nicotine-specific Ab responses.

### Anti-nicotine IgG ELISA

Anti-nicotine antibodies were quantified by ELISA using standard methods for endpoint titer determinations. A BSA-nic coating reagent was synthesized as described above using 100 molar equivalents of the Nic-6-HA-NHS ester. The spectrophotometric assay showed 32 of 34 lysines conjugated to nicotine. ELISA plates (NUNC) were incubated at 4°C overnight with BSA-Nic in 1X PBS (100 ng/well), blocked with 200 µL of 1X PBS +0.05%, v/v, Tween 20, 3% BSA at room temperature for 1 h and then washed 4x with 1X PBS +0.05%, v/v, Tween 20. Serial 10-fold dilutions of naïve and immunized mouse sera were prepared in blocking buffer and 100 ul of the samples were incubated in the ELISA plate for 1 hour. After washing, plates were incubated with rat anti-mouse IgG-HRP (Southern Biotech, New Orleans, LA) for 1 hour, washed and incubated with 100 µl of SureBlue TMB Microwell Peroxidase Substrate (KPL, Inc., Gaithersburg, MD) for 20 min. Development was stopped by adding 100 µl of TMB Stop solution (KPL, Inc., Gaithersburg, MD) and absorbance was measured at 450 nm with an automated plate reader to quantify HRP activity. Endpoint titer values were calculated as previously described [36].

### IgG Affinity by Competition ELISA

ELISA plates were coated with BSA-nic at 100 picograms per well in 1X PBS, incubated overnight at 4°C, and blocked as previously described. Mouse serum samples were diluted in blocking buffer to achieve absorbance values of approximately 1.0–1.5 at 450 nm following the HRP development assay. The antibody dilution used resulted in less than 5% of the nicotine specific IgG removed from the equilibrium solution (data not shown); Nicotine (Sigma-Aldrich, St. Louis, MO) was serially diluted from 0.0002–200 µM in blocking buffer. Equal volumes (50 µL) of diluted sera and nicotine were combined and incubated for 1 h at room temperature until equilibrium was reached. Serum/nicotine mixtures were added (100 µL/well) to the nicotine-BSA coated plates. The plates were incubated for 1 hour at room temperature and then washed as before. Thereafter, detection of antibody binding proceeded as outlined above for anti-nicotine antibody ELISA. Data were transformed according to the method of Friguet et al. [37] with the correction factor applied for bivalent IgG antigen binding described by Stevens et al. [38]. The Kd's determined by solid phase methodologies like competitive ELISA are relative values that may not be comparable to the absolute Kd values measured in solution by equilibrium dialysis.

### Serum Nicotine Binding Capacity

The function of anti-nicotine Ab was evaluated by measuring nicotine-binding capacity as previously described [39]. Serum collected from immunized mice on d160 was incubated for 2 hours at room temperature with 0.0001–10 µM nicotine. These samples were then subjected to equilibrium dialysis against an equal volume of 1X PBS for 16 hours at 4°C using a DispoEquilibrium DIALYZER cartridge, 10,000 MWCO (Harvard Apparatus, Holliston, MA). Aliquots from the buffer side of the dialysis membranes were removed and analyzed by SELDI-TOF MS (Bio-Rad, Hercules, CA) after immobilization onto a normal phase protein chip and addition of EAM-1 matrix. Unbound nicotine was quantified by comparing peak intensities to a standard curve generated with a nicotine standard.

### Nicotine Distribution in Brain and Sera

Anti-nicotine Abs were also evaluated for function by measuring nicotine distribution within brain and serum following anesthetization and tail vein infusion (<5 s) of 0.05 mg/kg of nicotine hydrogen tartrate diluted in 100 µL of PBS, which approximates the mg/kg dose of nicotine equivalent to three smoked cigarettes in humans [39]. Mice were sacrificed after 5 minutes. Blood was collected via cardiac puncture for serum preparation and the brain was removed, weighed and flash frozen in liquid nitrogen. Total nicotine in serum and brain tissue was measured by mass spectrophotometry (Alturus Inc, Moscow ID). In order to correct the brain nicotine content for the nicotine remaining in the brain's vasculature, we subtracted the total brain nicotine values by 4.4%, which is the estimated cerebral blood volume in mice, and adjusted the nicotine concentration in blood accordingly [40].

### Statistical analysis

Data were analyzed using GraphPad Prism (GraphPad Software, San Diego, CA). Statistical significance of the difference between two groups was calculated by Student's 2-tailed *t*-test on log-transformed data and between three or more groups by 1-factor analysis of variance (ANOVA) followed by post-hoc analysis. Differences were considered to be not significant with p>0.05.

## Results

### Hapten carrier design

**Figure 2:**
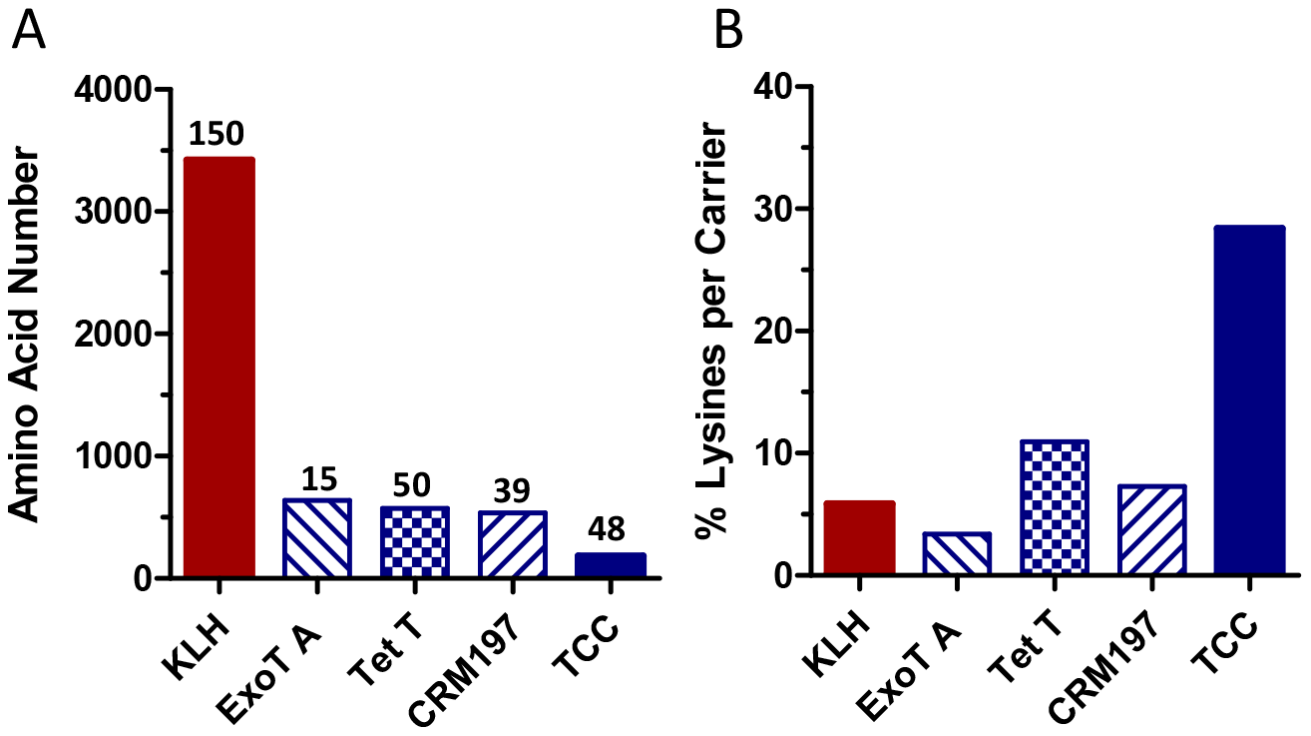
A comparison of the relative size and lysine content of the TCC and four hapten carriers; KLH, Exotoxin A (ExoT A), Tetanus toxoid (Tet T), and the inactivated diphtheria toxin protein CRM197; (A) The number of total amino acids with the number of lysines available for hapten conjugation reported above the bar; (B) The percentage of lysines in each carrier protein.

To investigate methods for improving functional antibody responses to drugs of abuse like nicotine, we designed and synthesized a trimeric coiled coil protein (TCC) containing three relatively short amphipathic α-helical peptides, each of which is followed by a CD4 T cell epitope (Fig. 1). The basic unit of each peptide is a repeating heptad sequence that alternates between 16 hydrophilic lysines and 9 hydrophobic isoleucines with an α-helical turn every 3.6 residues [41], [42]. Following TCC self-assembly, the repeating isoleucine residues form a hydrophobic core along the length of the carrier, and the charged lysines used for hapten conjugation become solvent-exposed along the helix surface. Arginine and glutamic acid residues within the repeating heptad peptide form salt bridges between adjacent helices to further stabilize the trimeric structure. This design creates a readily accessible conformation for hapten attachment and a series of B cell epitopes with defined spacing and stoichiometry. The C-terminal portion of each monomer peptide contains a CD4 T cell epitope required for the production of long-lived plasma cells and high affinity antibodies [24], [25]. Following conjugation, this multivalent antigen contains a much higher density of haptens as compared to other carrier proteins, which is a key determinant of immunogenicity [14]–[20]. Fig. 2 compares the relative size and lysine content of the TCC with 4 different nicotine-hapten carriers; KLH is a commonly used hapten carrier protein; Exotoxin A is the carrier for NicVax that failed in two Phase III clinical studies [10]; Tetanus toxoid (Niccine) failed in a Phase II smoking relapse study [13], and the diphtheria toxin protein CRM197 is currently being tested in a Phase I study. The number of haptens conjugated to these clinical-stage carriers has averaged between 10–30 haptens per protein [43].

### TCCnic immunogenicity

**Figure 3:**
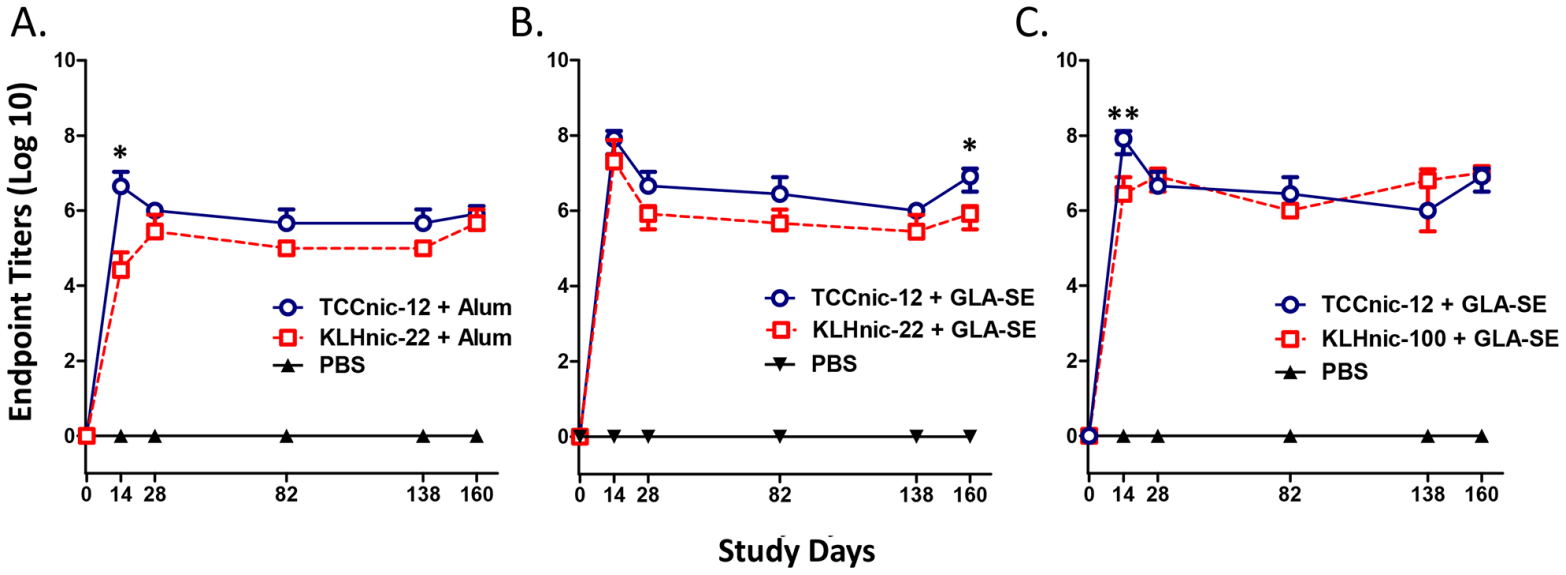
Anti-nicotine antibody responses in immunized mice. C57BL/6 mice (5/grp) were injected (d0, d14, d131) with either PBS or 2.5 µg of the indicated conjugate hapten carriers and adjuvants, and sera was assayed for anti-nicotine Ab titers by ELISA. TCCnic-12 contained an average 12 haptens per trimer, whereas KLHnic-22 and KLHnic-100 contained, respectively an average 22 and 100 haptens per monomer protein. Comparisons between groups were conducted by unpaired two-tailed *t*-test; *p<0.004; **p<0.002.

**Figure 4:**
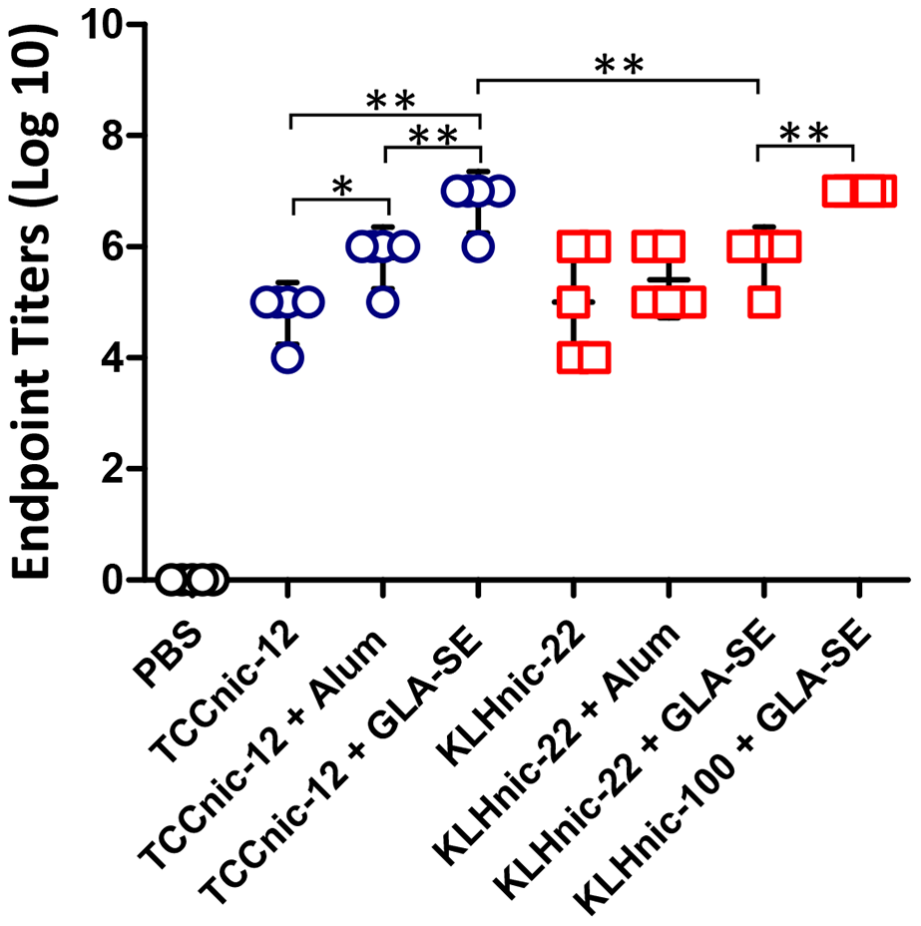
Day 160 anti-nicotine Ab titers. C57BL/6 mice (5/grp) were immunized (d0, d14, d146) with either PBS or 2.5 µg of the indicated conjugated hapten carriers and adjuvants, and serum was assayed by ELISA. Comparisons between groups were conducted by unpaired two-tailed *t*-test; *p<0.003; **p<0.0001.

To characterize antibody responses using C57BL/6 mice, we synthesized TCC containing two H2D^b^ restricted helper T-cell epitopes; the 13 AA PADRE sequence [44], followed by an 11 AA sequence present in the H5N1 hemagglutinin [26]. TCC was conjugated with a nicotine derivative containing hexanoic acid at the 6 position of the pyridine ring (Nic-6-HA), with the final construct averaging 12 haptens per trimer (TCCnic-12). We also prepared 2 conjugated KLH carriers as controls. The first, KLHnic-22, contained an average 22 nicotines per monomer, which approximates a hapten loading similar to many preclinical and clinical vaccines [44]. The second, KLHnic-100, was a hyper-conjugated carrier compared to most nicotine vaccines that was used to test the impact of increased hapten density on vaccine immunogenicity. Separately, to measure the role that adjuvants play on vaccine function, we formulated these carriers with either Alum or GLA-SE. C57BL/6 mice (5/grp) were immunized three times (d0, d14, and d131) and serum was assayed for anti-nicotine Ab titers by ELISA. The kinetics of the resulting Ab responses is presented in Fig. 3. As indicated, all three carriers stimulated Abs titers that remained near maximal levels throughout the course of the experiment. The titers induced by TCCnic-12 adjuvanted with either Alum or GLA-SE appeared slightly greater than adjuvanted KLHnic-22 over the course of the experiment (Figs. 3A and 3B), while the activities of TCCnic-12 and KLHnic100 adjuvanted with GLA-SE were the same (Fig. 3C). Fig. 4 presents the d160 endpoint titers that were collected 3 weeks following the final boost injection. With respect to adjuvant activity, mice immunized with TCCnic-12 + GLA-SE stimulated an Ab response that was ∼100x better than TCCnic-12 alone and ∼10x greater than TCCnic-12+ Alum. GLA-SE also improved the responses rates of mice immunized with KLHnic-22, but the differences in mean titers were not statistically significant. With regards to the hapten carriers, TCCnic-12+ GLA-SE stimulated ∼10x more antibody than KLHnic-22 + GLA-SE, while TCCnic-12 and KLHnic-100 activities were equivalent in the presence of GLA-SE. In addition, KLHnic-100 + GLA-SE induced consistently greater Ab titers than KLHnic-22 + GLA-SE throughout the experiment. Collectively, these results demonstrate that the TCC hapten carrier can effectively stimulate anti-nicotine Ab responses in mice. They also confirm previous studies that hapten density is an important variable for conjugate vaccine immunogenicity.

**Figure 5:**
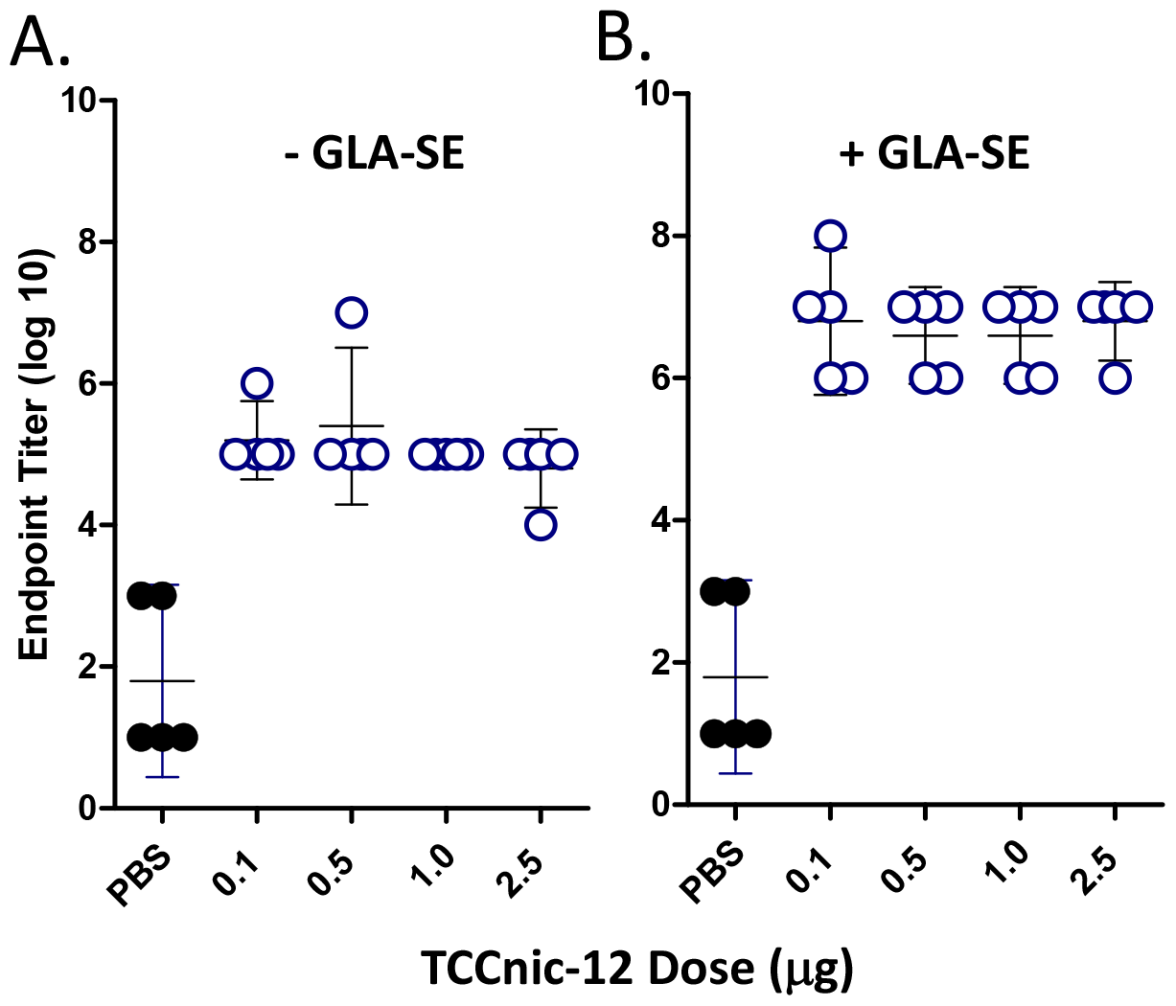
Vaccine dose response. C57BL/6 mice (5/grp) were immunized with the indicated doses of TCCnic-12 in the absence (A) and presence (B) of GLA-SE, and d35 serum was assayed for anti-nicotine Ab by ELISA.

**Figure 6:**
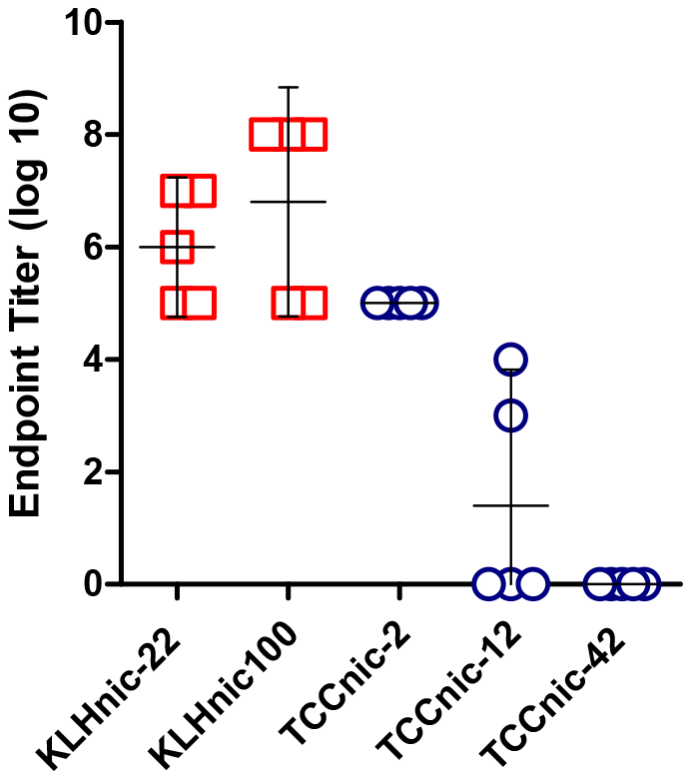
Anti-carrier Ab responses in immunized mice. C57BL/6 mice (5/grp) were injected (d0, d14) with the indicated carriers in the presence of GLA-SE. TCCnic-2, TCCnic-12, and TCCnic-42 contained, respectively, an average 2, 12, and 42 haptens per trimer. Day 28 serum from each group was assayed for Ab that bound the corresponding *unconjugated* hapten carrier.

In a follow-on experiment, we measured the dose response of TCCnic-12 +/− GLA-SE and determined that the lowest antigen dose (100 ng) induced the maximal Ab titers (Fig. 5). This result suggests that the full complement of lymphocytes capable of responding to the hapten carrier were primed by the conjugate and that the presence of adjuvant augmented clonal expansion and the downstream effector phase of the response. In a separate experiment, we tested whether the TCC induced anti-carrier Abs similar to a conventional nicotine vaccine. In this experiment, mice were immunized with the controls, KLHnic-22 and KLHnic-100, along with a TCC that was conjugated with an average of 2, 12, or 42 haptens per trimer. As indicated in Fig. 6, the induction of anti-TCC Abs relative to anti-KHL titers was markedly diminished with increasing hapten density. This result demonstrates the importance of hapten density in controlling anti-carrier responses and suggests that TCC may be less likely to induce neutralizing Abs relative to current nicotine carriers.

### TCCnic functional antibody responses

**Figure 7:**
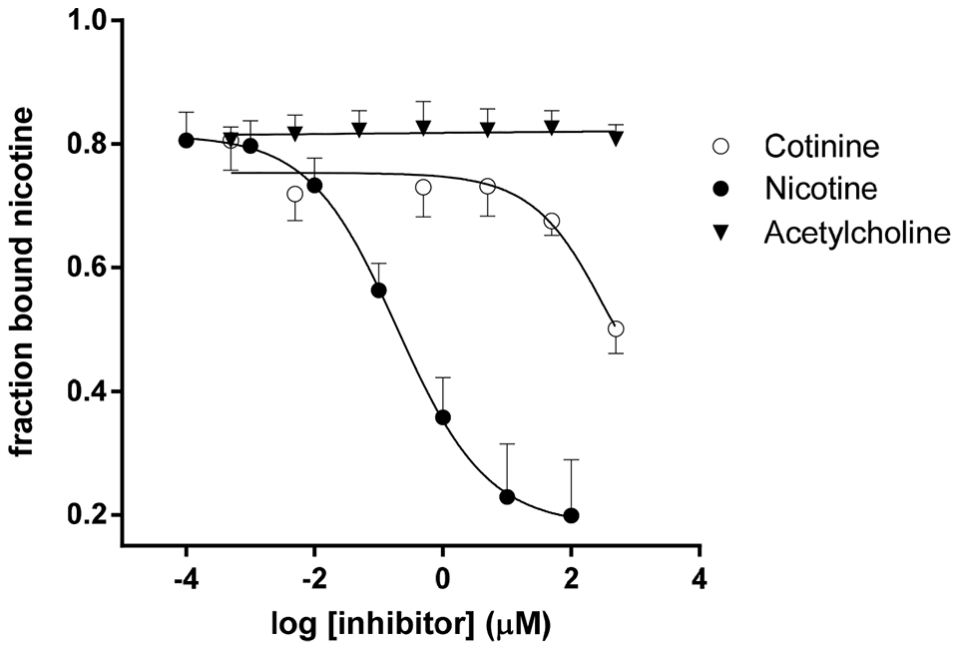
Antibody specificity. The specificity of nicotine binding to antisera collected from TCCnic-12 immunized mice was determined by competitive ELISA for nicotine, cotinine and acetylcholine. IC_50_ values for cotinine were 1000-fold greater than nicotine and could not be calculated for acetylcholine due to a lack of inhibition.

**Figure 8:**
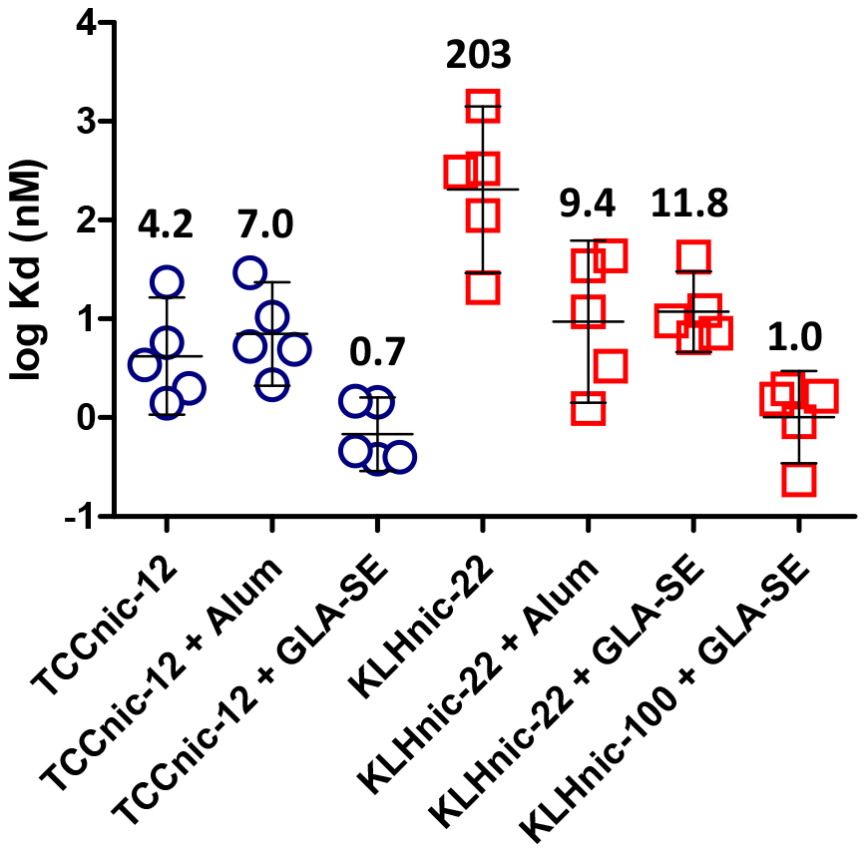
Relative affinities of anti-nicotine Abs induced by TCCnic-12, KLHnic-22, and KLHnic-100, either alone or in the presence of Alum or GLA-SE. C57BL/6 mice (5/grp) were injected (d0, d14, d146) with either PBS or 2.5 µg of the indicated conjugate hapten carriers and adjuvants. The geometric mean Kd values indicated above each data set were determined by competitive ELISA.

In addition to Ab titers, we examined the quality of the Abs induced with TCCnic-12. As shown in Fig. 7, these antibodies were highly specific to nicotine and did not bind physiological concentrations of cotinine, the most abundant metabolite in the nicotine degradation pathway, nor acetylcholine, the endogenous nicotine receptor ligand. Similar results were obtained with KLHnic-22 and KLHnic-100, and no differences in specificity were seen with either adjuvant (data not shown). The affinity of these antibodies were also measured (Fig. 8). As indicated by the relative differences in Kd values, non-adjuvanted TCCnic-12 induced Abs with a much higher affinity (4.2 nM) than KLHnic-22 (203 nM). The addition of Alum had no apparent impact on the TCCnic-12 response, although it did improve KLHnic-22 antibody affinity (9.4 nM). In the presence of GLA-SE, Ab affinity in mice immunized with TCCnic-12 increased even further (0.7 nM), and was an order of magnitude greater than the KLHnic-22 + GLA-SE response (11.8 nM). The affinity of the nicotine Abs induced in mice immunized with KLHnic100 + GLA-SE (1.0 nM) was also greater than KLHnic-22 + GLA-SE and was equivalent to TCCnic-12 + GLA-SE.

**Figure 9:**
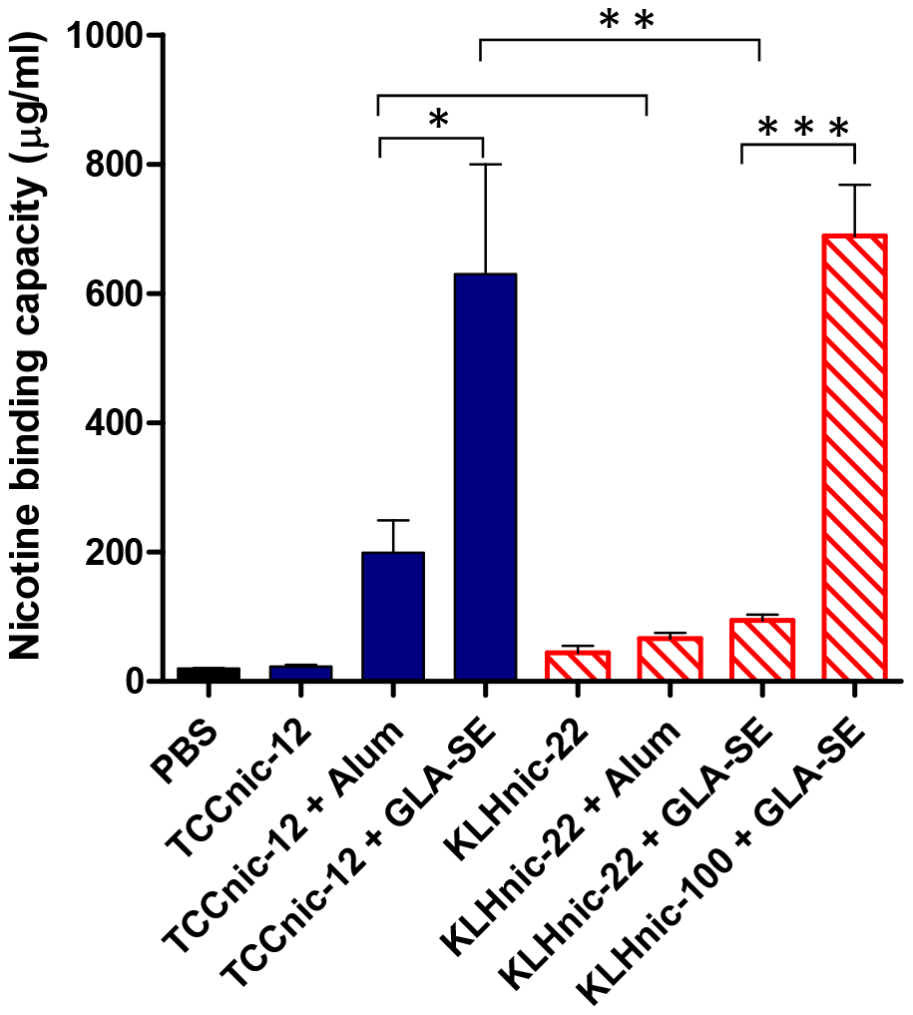
Serum nicotine binding capacity was determined by measuring bound and free concentrations of nicotine at equilibrium. Kd values (Fig. 8) were used to calculate total antibody concentrations according to the law of mass action equation: Kd  =  [Nic][IgG]/[Nic-IgG]. Comparisons between groups were conducted by unpaired two-tailed *t*-test; *p<0.04; **p<0.01; ***p<0001.

**Figure 10:**
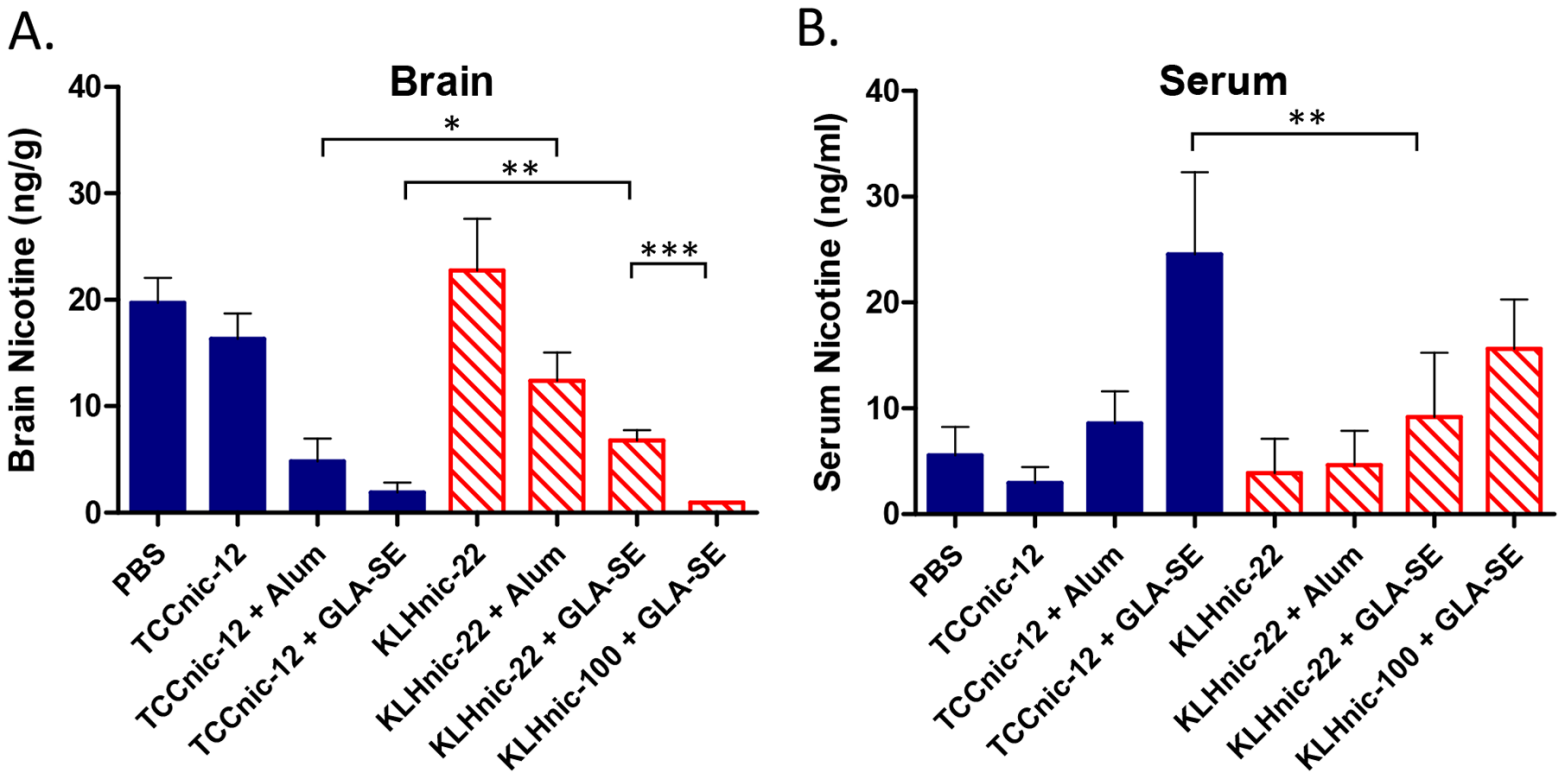
Anti-nicotine Ab function in mice. C57BL/6 mice (5/grp) were injected (d0, d14, d146) with either PBS or 2.5 ug of the indicated conjugate hapten carriers and adjuvants. Mice (5/grp) were injected on d160 with a dose of nicotine tartrate equivalent to 3 cigarettes (1.2 ug). Five minutes later the mice were sacrificed, tissues removed and the amounts of nicotine in brain (A); and serum (B) were measured by mass spectrometry; * p<0.05; ** p<0.007; *** p<0.0003.

To measure antibody function, we first determined the nicotine binding capacity within the sera of immunized mice. As shown in Fig. 9, TCCnic-12 + GLA-SE and KLHnic-100 + GLA-SE induced the largest binding capacities which, as expected, correlated with their respective antibody titers (Fig. 3) and affinities (Fig. 8). As a second measure of Ab function, we injected immunized mice with a dose of nicotine equivalent to 3 cigarettes (0.05 mg/kg), and after 5 minutes used mass spectrometry to quantify the amount of nicotine that had accumulated in brain tissue (Fig. 10). Again, the best performing vaccines were TCCnic-12 + GLA-SE and KLHnic100 + GLA-SE where nicotine entry into the brain was inhibited by, respectively, 91% and 95% relative to the PBS control animals. The degree of inhibition for the other constructs was 76% for TCCnic-12 + Alum, 62% for KLHnic-22 + GLA-SE, and 47% for KLHnic-22 + Alum. TCCnic-12 stimulated a superior response compared to KLHnic-22 when adjuvanted with either Alum or GLA-SE, and KLHnic-100 + GLA-SE out-performed KLHnic-22 + GLA-SE. Collectively, these results demonstrate that TCCnic-12 is an effective hapten carrier for inducing functional Ab titers in mice. These findings also demonstrate that hapten density and the quality of the adjuvant play important roles in regulating nicotine vaccine function.

## Discussion

Clinical findings suggest that the vaccines tested for smoking cessation failed to induce meaningful titers of functional Abs sufficient to block nicotine entry into brain [12]. A number of variables are known to influence conjugate vaccine performance including the carrier, hapten structure, hapten density, and the choice of adjuvant [39], [43]. To help characterize and improve vaccine activity, we have constructed a chemically-defined synthetic hapten carrier that is short in length, has very low sequence complexity, and due to its coiled-coil structure, is highly stable [41], [42]. This design creates a readily accessible conformation for hapten conjugation, and in contrast to native protein carriers, a series of identical B cell epitopes with defined spacing and stoichiometry that should facilitate the identification of optimal hapten structures and their densities. In addition, the placement of one or more defined CD4 T cell epitopes at the ends of each monomer of the coil creates the opportunity for a personalized vaccine that could simultaneously improve individual antibody responses and diminish the large variability in serum Ab titers seen in the clinic.

As an initial test, we measured the functional activity of a TCC carrier containing an average of 12 nicotine haptens in the presence and absence of two different adjuvants, and compared this activity to a traditional carrier that differed in hapten loading by 5-fold. The results clearly showed that the TCC could induce large titers of high affinity anti-nicotine antibodies in mice, which in turn, generated a nicotine binding capacity sufficient to prevent 90% of a relevant dose of nicotine from reaching the brain. They also argue that the TCC may be effective for human use because of its increased epitope density, which is known to control antigen binding, B cell activation, IgG production, and Ab affinity [14]–[20]. This conclusion is based on the close correlation between the differences in Ab titer (Figs. 3 and 4), affinity (Fig. 8), and functional activity (Figs. 9 and 10) measured between the vaccines and the relative percentage of haptens per carrier, which was 6.5% for TCCnic-12, 3% for KLHnic-100, and 0.6% for KLHnic-22. Future studies will test how the relative hapten load on the TCC impacts vaccine function.

In a separate experiment, we measured the Ab response to the TCC carrier, and observed that relative to anti-KLH titers, Abs directed to the TCC were increasingly diminished with increasing hapten density. While we did not test neutralizing activity, this result suggests that the TCC may be less likely to induce epitope suppression relative to current carriers. It was also seen that maximum anti-nicotine Ab titers were induced with as little as 100 ng of the conjugated TCC, which was 25 times lower than the initial starting dose. This argues that the lymphocytes capable of recognizing the 6-nic-HA hapten were activated at a relatively small dose of the vaccine, above which has no positive effect on nicotine Ab titer and might actually be detrimental with respect to anti-carrier responses. Recent studies have shown that co-immunization of dissimilar nicotine haptens produced additive antibody responses [45], [46]. This provides a strong rationale for testing a multivalent TCC-based vaccine that could activate numerous B cell populations and augment functional Ab responses even further.

The synthetic nature of a TCC-based vaccine provides significant advantages over a recombinant protein carrier with respect to clinical development and commercialization [47]. For instance, cGMP manufacturing using solid-phase synthesis is quicker and yields a highly refined protein with fewer regulatory concerns. Increasing carrier production levels from gram to kg amounts would be considerably less expensive and require limited comparability and revalidation studies. Another advantage is that the TCC can be conjugated with the nicotine hapten in situ during solid phase synthesis, whereas recombinant protein conjugation produces a more heterogeneous product requiring a more complicated purification and scale-up process. The superiority of synthetic carrier manufacturing becomes even more apparent for production of a multivalent vaccine, where again, multiple TCC-based antigens could be produced reproducibly during a single round of synthesis, while a multivalent recombinant vaccine would require development of multiple independent conjugation, purification, and confirmation procedures, which magnifies the time and costs even further.

Vaccine adjuvants control the magnitude and quality of adaptive T and B cell responses by facilitating antigen uptake into antigen presenting cells and stimulating innate pathways that control leukocyte recruitment to the site of injection [48]. To date, the only adjuvant used in clinical nicotine vaccine studies has been Alum, however numerous studies suggests that Alum may be relatively weak in comparison to adjuvants that target innate pattern recognition receptors on APC [48]. The receptor that binds bacterial LPS, TLR-4, plays a critical role in CD4 T cell regulation of germinal center formation, affinity maturation, and the production of long-lived antibody-secreting plasma cells [49]–[51], and we have shown previously that adjuvants formulated with the synthetic TLR-4 ligand, GLA, are potent stimulators of protective T-cell mediated antibody responses against heterosubtypic H5N1 influenza viruses [26], [52]. Here we learned that, relative to an Alum adjuvant, GLA-SE played a major role in regulating higher Ab titers, improved Ab affinities, and a significant increase in functional inhibitor activity. The observation that GLA-mediated antibody responses were larger and more consistent with TCCnic-12 than KLHnic-22 may result from the placement of 2 dominant H2D^b^ restricted helper T-cell epitopes within each monomer of the TCC. In summary, we have developed two important tools that could significantly improve the performance of anti-addiction vaccines in people. The first is a novel synthetic hapten carrier and the second is the adjuvant GLA-SE, which was far superior to Alum in augmenting anti-nicotine Ab titer, affinity, and function. This is consistent with previous work showing that addition of the TLR9 ligand CpG to Alum significantly improved functional nicotine antibody responses in both mice and Cynomolgus monkeys [39], [53].
