# Correction: Novel Anti-Nicotine Vaccine Using a Trimeric Coiled-Coil Hapten Carrier

**DOI:** 10.1371/journal.pone.0122506

**Published:** 2015-03-25

**Authors:** The PLOS ONE Staff

Fig. 9 is incorrect. The unit used in the y-axis should be ng/ml instead of μg/ml. The authors have provided a corrected Fig. 9 here.

**Fig 9:**
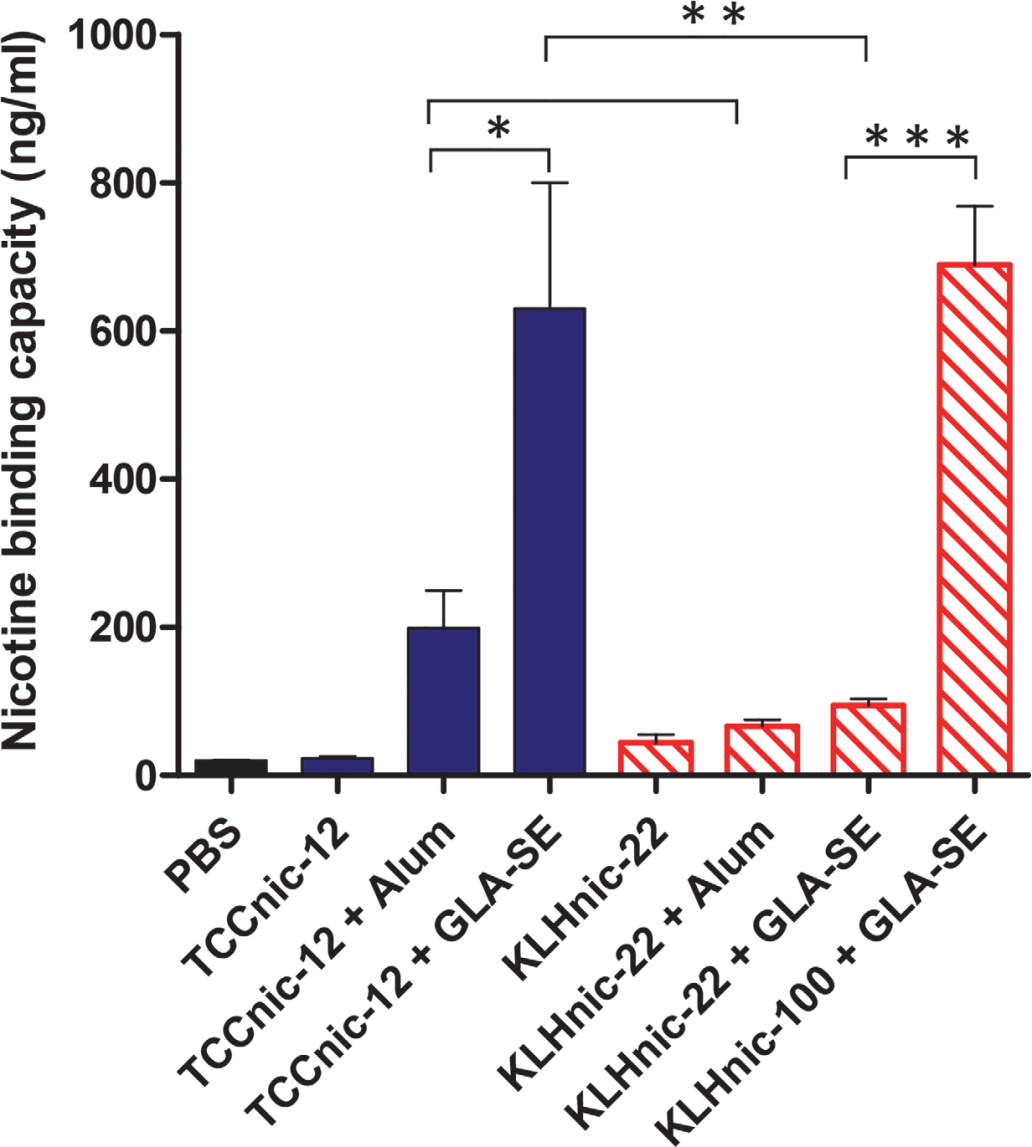
Serum nicotine binding capacity was determined by measuring bound and free concentrations of nicotine at equilibrium. Kd values (Fig. 8) were used to calculate total antibody concentrations according to the law of mass action equation: Kd = [Nic][IgG]/[Nic-IgG]. Comparisons between groups were conducted by unpaired two-tailed t-test; *p<0.04; **p<0.01; ***p<0001.
